# Crystal structure of tri­aqua­(1,10-phen­anthroline-κ^2^ *N*,*N*′)(2,4,5-tri­fluoro-3-meth­oxy­benzoato-κ*O* ^1^)cobalt(II) 2,4,5-tri­fluoro-3-meth­oxy­benzoate

**DOI:** 10.1107/S1600536814022077

**Published:** 2014-10-11

**Authors:** Junshan Sun

**Affiliations:** a Beijing Key Laboratory for Science and Application of Functional Molecular and Crystalline Materials, Department of Chemistry, University of Science and Technology Beijing, Beijing,100083, People’s Republic of China

**Keywords:** crystal structure, cobalt(II) complex, phenanthroline ligands, 2,4,5-tri­fluoro-3-meth­oxy­benzoate ligands, hydrogen bonding

## Abstract

The title salt, [Co(C_8_H_4_F_3_O_3_)(C_12_H_8_N_2_)(H_2_O)_3_](C_8_H_4_F_3_O_3_), was obtained under solvothermal conditions by the reaction of 2,4,5-tri­fluoro-3-meth­oxy­benzoic acid with CoCl_2_ in the presence of 1,10-phenanthroline (phen). The Co^II^ ion is octa­hedrally coordinated by two N atoms [Co—N = 2.165 (2) and 2.129 (2) Å] from the phen ligand, by one carboxyl­ate O atom [Co—O = 2.107 (1) Å] and by three O atoms from water mol­ecules [Co—O = 2.093 (1), 2.102 (1) and 2.114 (1) Å]. The equatorial positions of the slightly distorted octa­hedron are occupied by the N atoms, the carboxyl­ate O and one water O atom. An intra- and inter­molecular O—H⋯O hydrogen-bonding network between the water-containing complex cation and the organic anion leads to the formation of ribbons parallel to [010].

## Related literature   

For complexes of Co^II^, see: Wang *et al.* (2008 ▶); Li *et al.* (2014 ▶). For metal cations chelated by phenanthroline or its derivatives, see: Liu *et al.* (2006 ▶); Kaizer *et al.* (2006 ▶).
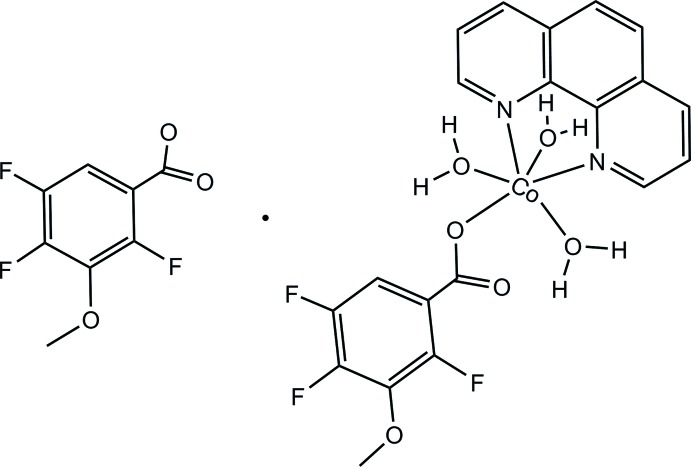


## Experimental   

### Crystal data  

[Co(C_8_H_4_F_3_O_3_)(C_12_H_8_N_2_)(H_2_O)_3_](C_8_H_4_F_3_O_3_)
*M* *_r_* = 703.41
Monoclinic, 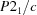

*a* = 17.177 (3) Å
*b* = 7.0429 (14) Å
*c* = 26.543 (4) Å
β = 116.942 (9)°
*V* = 2862.6 (9) Å^3^
*Z* = 4
Mo *K*α radiation
μ = 0.70 mm^−1^
*T* = 273 K
0.31 × 0.24 × 0.22 mm

### Data collection  

Bruker SMART CCD diffractometer
Absorption correction: multi-scan (*SADABS*; Bruker, 2005 ▶) *T*_min_ = 0.813, *T*_max_ = 0.862
14578 measured reflections
5074 independent reflections
4221 reflections with *I* > 2σ(*I*)
*R*_int_ = 0.031

### Refinement  

*R*[*F* ^2^ > 2σ(*F* ^2^)] = 0.031
*wR*(*F* ^2^) = 0.083
*S* = 1.05
5074 reflections
417 parameters
H-atom parameters constrained
Δρ_max_ = 0.32 e Å^−3^
Δρ_min_ = −0.29 e Å^−3^

### 

Data collection: *SMART* (Bruker, 2005 ▶); cell refinement: *SAINT* (Bruker, 2005 ▶); data reduction: *SAINT*; program(s) used to solve structure: *SHELXS97* (Sheldrick, 2008 ▶); program(s) used to refine structure: *SHELXL97* (Sheldrick, 2008 ▶); molecular graphics: *SHELXTL* (Sheldrick, 2008 ▶); software used to prepare material for publication: *SHELXTL*.

## Supplementary Material

Crystal structure: contains datablock(s) I, global. DOI: 10.1107/S1600536814022077/wm5067sup1.cif

Structure factors: contains datablock(s) I. DOI: 10.1107/S1600536814022077/wm5067Isup2.hkl

**Fig. 1.**
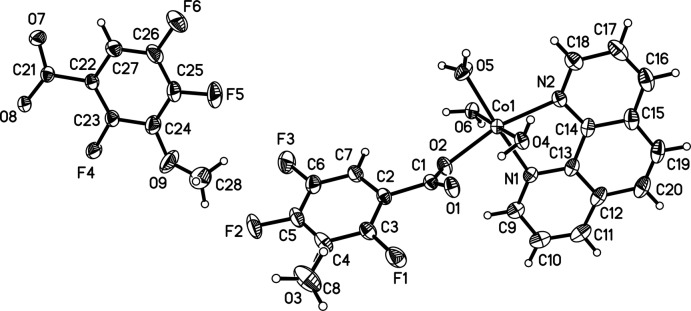
The molecular structure of title compound, with at1om labels and displacement ellipsoids drawn at the 30% probability level.

. DOI: 10.1107/S1600536814022077/wm5067fig1.tif
The mol­ecular structure of title compound, with atom labels and displacement ellipsoids drawn at the 30% probability level.

**Fig. 2.**
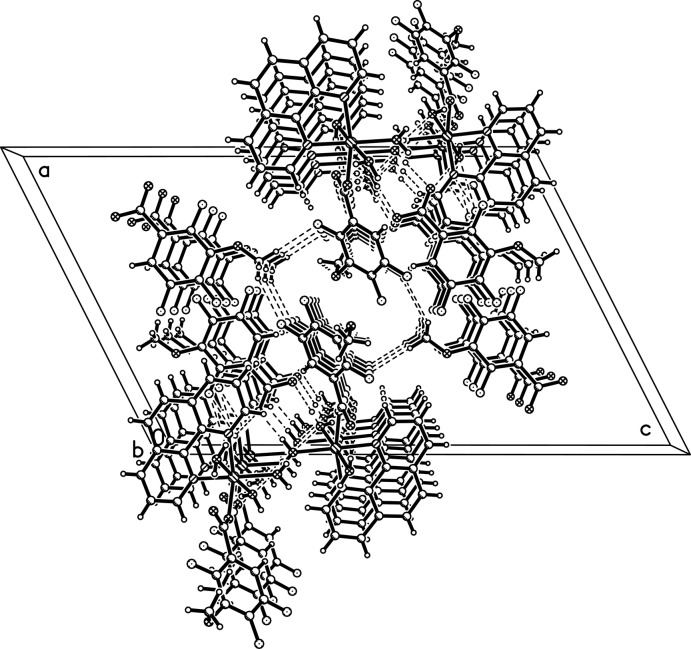
The crystal packing of title compound. Hydrogen bonds are shown by dashed lines.

. DOI: 10.1107/S1600536814022077/wm5067fig2.tif
The crystal packing of title compound. Hydrogen bonds are shown by dashed lines.

CCDC reference: 1027800

Additional supporting information:  crystallographic information; 3D view; checkCIF report

## Figures and Tables

**Table 1:** Hydrogen-bond geometry (, )

*D*H*A*	*D*H	H*A*	*D* *A*	*D*H*A*
O4H4*A*O1	0.85	1.78	2.604(2)	163
O4H4*B*O8^i^	0.85	1.89	2.729(2)	169
O5H5*A*O8^ii^	0.85	1.94	2.791(2)	176
O5H5*B*O6^iii^	0.85	2.15	2.976(2)	165
O6H6*A*O1^iv^	0.85	1.90	2.738(2)	167
O6H6*B*O7^ii^	0.85	1.77	2.623(2)	176

Symmetry codes: (i) 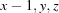; (ii) 
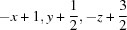
; (iii) 
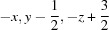
; (iv) 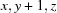.

## supplementary crystallographic information

### S1. Experimental

The reaction was carried out under solvothermal conditions. 2,4,5-Trifluoro-3-methoxyl benzoic acid (0.220 g, 1 mmol), CoCl_2_ (0.130 g, 1 mmol) and phenanthroline (0.156 g, 1 mmol) were added to an airtight vessel together with methanol and water in a ratio of 1:2 (*v*/*v*). The vessel was heated at 393 K for three days and was then cooled down to room temperature with a rate of 10 Kh^-1^. The resulting blue solution was filtered. The filtrate was placed for several days yielding blue block-shaped crystals in a yield of 78%. Elemental analysis: calc. for C_28_H_22_CoF_6_N_2_O_9_: C 47.81, H 3.15, N 3.98; found: C 47.50, H 3.47, N 3.62. The elemental analyses were performed with a Perkin-Elmer model 2400 series II.

### S2. Refinement

The water H atoms could be located in difference Fourier maps and were refined with a distance O–H of 0.85 Å, the aromatic H atoms were placed in calculated positions. The *U*_iso_(H) values were set at 1.2*U*_eq_(C) for the aromatic H atoms, and 1.5*U*_eq_(O) for water molecules.

### Crystal data

**Table:**

[Co(C_8_H_4_F_3_O_3_)(C_12_H_8_N_2_)(H_2_O)_3_](C_8_H_4_F_3_O_3_)	*F*(000) = 1428
*M**_r_* = 703.41	*D*_x_ = 1.632 Mg m^−^^3^
Monoclinic, *P*2_1_/*c*	Mo *K*α radiation, λ = 0.71073 Å
Hall symbol: -P 2ybc	Cell parameters from 6632 reflections
*a* = 17.177 (3) Å	θ = 2.4–27.5°
*b* = 7.0429 (14) Å	µ = 0.70 mm^−^^1^
*c* = 26.543 (4) Å	*T* = 273 K
β = 116.942 (9)°	Block, blue
*V* = 2862.6 (9) Å^3^	0.31 × 0.24 × 0.22 mm
*Z* = 4	

### Data collection

**Table:**

Bruker SMART CCD diffractometer	5074 independent reflections
Radiation source: fine-focus sealed tube	4221 reflections with *I* > 2σ(*I*)
Graphite monochromator	*R*_int_ = 0.031
ω scans	θ_max_ = 25.1°, θ_min_ = 1.7°
Absorption correction: multi-scan (*SADABS*; Bruker, 2005)	*h* = −18→20
*T*_min_ = 0.813, *T*_max_ = 0.862	*k* = −8→8
14578 measured reflections	*l* = −30→31

### Refinement

**Table:**

Refinement on *F*^2^	Primary atom site location: structure-invariant direct methods
Least-squares matrix: full	Secondary atom site location: difference Fourier map
*R*[*F*^2^ > 2σ(*F*^2^)] = 0.031	Hydrogen site location: inferred from neighbouring sites
*wR*(*F*^2^) = 0.083	H-atom parameters constrained
*S* = 1.05	*w* = 1/[σ^2^(*F*_o_^2^) + (0.0434*P*)^2^ + 0.4144*P*] where *P* = (*F*_o_^2^ + 2*F*_c_^2^)/3
5074 reflections	(Δ/σ)_max_ = 0.055
417 parameters	Δρ_max_ = 0.32 e Å^−^^3^
0 restraints	Δρ_min_ = −0.29 e Å^−^^3^

### Special details

**Table:**

Geometry. All esds (except the esd in the dihedral angle between two l.s. planes) are estimated using the full covariance matrix. The cell esds are taken into account individually in the estimation of esds in distances, angles and torsion angles; correlations between esds in cell parameters are only used when they are defined by crystal symmetry. An approximate (isotropic) treatment of cell esds is used for estimating esds involving l.s. planes.
Refinement. Refinement of *F*^2^ against ALL reflections. The weighted *R*-factor *wR* and goodness of fit *S* are based on *F*^2^, conventional *R*-factors *R* are based on *F*, with *F* set to zero for negative *F*^2^. The threshold expression of *F*^2^ > σ(*F*^2^) is used only for calculating *R*-factors(gt) *etc*. and is not relevant to the choice of reflections for refinement. *R*-factors based on *F*^2^ are statistically about twice as large as those based on *F*, and *R*- factors based on ALL data will be even larger.

### Fractional atomic coordinates and isotropic or equivalent isotropic displacement parameters (Å^2^)

**Table:**

	*x*	*y*	*z*	*U*_iso_*/*U*_eq_	
Co1	0.036012 (16)	0.98486 (3)	0.849126 (11)	0.03250 (10)	
F1	0.26524 (9)	0.3645 (2)	0.97664 (7)	0.0707 (4)	
F2	0.50025 (8)	0.4048 (2)	0.93279 (7)	0.0792 (5)	
F3	0.42723 (10)	0.7110 (3)	0.86781 (7)	0.0915 (6)	
O1	0.11946 (9)	0.54591 (19)	0.90080 (7)	0.0471 (4)	
O2	0.15660 (8)	0.84291 (19)	0.89122 (6)	0.0438 (3)	
O3	0.42421 (11)	0.2330 (2)	0.99177 (9)	0.0785 (6)	
O4	−0.02612 (8)	0.73632 (18)	0.85611 (6)	0.0400 (3)	
H4A	0.0146	0.6550	0.8702	0.060*	
H4B	−0.0695	0.6930	0.8271	0.060*	
O5	0.02512 (10)	0.9156 (2)	0.76868 (6)	0.0559 (4)	
H5A	0.0644	0.9599	0.7609	0.084*	
H5B	−0.0091	0.8439	0.7422	0.084*	
O6	0.09820 (9)	1.22487 (18)	0.83776 (6)	0.0396 (3)	
H6A	0.1083	1.3132	0.8616	0.059*	
H6B	0.1455	1.1992	0.8362	0.059*	
N1	0.04641 (11)	1.1074 (2)	0.92526 (7)	0.0369 (4)	
N2	−0.09265 (11)	1.1051 (2)	0.82265 (7)	0.0398 (4)	
C1	0.17226 (12)	0.6698 (3)	0.90106 (8)	0.0358 (4)	
C2	0.26251 (12)	0.6034 (3)	0.91315 (9)	0.0376 (5)	
C3	0.30280 (14)	0.4504 (3)	0.94752 (10)	0.0455 (5)	
C4	0.38301 (14)	0.3792 (3)	0.95534 (10)	0.0526 (6)	
C5	0.42300 (14)	0.4690 (3)	0.92756 (10)	0.0528 (6)	
C6	0.38504 (15)	0.6250 (4)	0.89423 (10)	0.0559 (6)	
C7	0.30619 (13)	0.6933 (3)	0.88676 (9)	0.0461 (5)	
H7	0.2818	0.7993	0.8642	0.055*	
C8	0.3839 (2)	0.0534 (4)	0.97619 (15)	0.0901 (10)	
H8A	0.3280	0.0668	0.9438	0.135*	
H8B	0.3763	0.0010	1.0071	0.135*	
H8C	0.4200	−0.0298	0.9671	0.135*	
C9	0.11689 (15)	1.1166 (3)	0.97473 (9)	0.0464 (5)	
H9	0.1694	1.0716	0.9772	0.056*	
C10	0.11616 (19)	1.1904 (3)	1.02325 (10)	0.0585 (7)	
H10	0.1674	1.1971	1.0570	0.070*	
C11	0.0396 (2)	1.2524 (3)	1.02041 (11)	0.0605 (7)	
H11	0.0379	1.2988	1.0527	0.073*	
C12	−0.03715 (17)	1.2468 (3)	0.96895 (10)	0.0502 (6)	
C13	−0.02984 (14)	1.1751 (3)	0.92162 (9)	0.0394 (5)	
C14	−0.10486 (14)	1.1669 (3)	0.86735 (9)	0.0409 (5)	
C15	−0.18697 (15)	1.2216 (3)	0.86204 (12)	0.0547 (6)	
C16	−0.25805 (17)	1.2051 (4)	0.80864 (14)	0.0686 (8)	
H16	−0.3137	1.2370	0.8034	0.082*	
C17	−0.24616 (15)	1.1424 (4)	0.76409 (13)	0.0640 (7)	
H17	−0.2934	1.1314	0.7284	0.077*	
C18	−0.16170 (15)	1.0947 (3)	0.77263 (10)	0.0521 (6)	
H18	−0.1540	1.0541	0.7418	0.063*	
C19	−0.1918 (2)	1.2905 (3)	0.91190 (16)	0.0704 (8)	
H19	−0.2458	1.3263	0.9090	0.084*	
C20	−0.1211 (2)	1.3044 (3)	0.96194 (14)	0.0669 (8)	
H20	−0.1267	1.3526	0.9928	0.080*	
F4	0.79608 (8)	0.6899 (2)	0.83581 (5)	0.0640 (4)	
F5	0.49409 (10)	0.7502 (3)	0.77106 (9)	0.1131 (7)	
F6	0.46705 (9)	0.6963 (3)	0.66524 (8)	0.1035 (6)	
O7	0.75824 (10)	0.6564 (3)	0.67096 (7)	0.0624 (5)	
O8	0.85075 (9)	0.5774 (2)	0.75883 (6)	0.0494 (4)	
O9	0.66903 (14)	0.7478 (5)	0.86077 (9)	0.1250 (11)	
C21	0.77750 (13)	0.6287 (3)	0.72160 (9)	0.0398 (5)	
C22	0.70364 (13)	0.6598 (3)	0.73757 (9)	0.0378 (5)	
C23	0.71580 (13)	0.6863 (3)	0.79192 (9)	0.0443 (5)	
C24	0.64763 (16)	0.7167 (4)	0.80576 (11)	0.0632 (7)	
C25	0.56427 (16)	0.7200 (4)	0.76156 (12)	0.0668 (7)	
C26	0.55010 (14)	0.6927 (4)	0.70685 (12)	0.0636 (7)	
C27	0.61828 (14)	0.6647 (3)	0.69431 (10)	0.0531 (6)	
H27	0.6077	0.6489	0.6569	0.064*	
C28	0.6204 (3)	0.7241 (9)	0.88452 (16)	0.169 (3)	
H28A	0.5735	0.8145	0.8699	0.253*	
H28B	0.6540	0.7421	0.9245	0.253*	
H28C	0.5969	0.5978	0.8772	0.253*	

### Atomic displacement parameters (Å^2^)

**Table:**

	*U*^11^	*U*^22^	*U*^33^	*U*^12^	*U*^13^	*U*^23^
Co1	0.03164 (16)	0.03054 (15)	0.03758 (16)	−0.00071 (10)	0.01767 (12)	−0.00163 (11)
F1	0.0532 (8)	0.0698 (10)	0.0854 (11)	0.0073 (7)	0.0282 (8)	0.0347 (8)
F2	0.0357 (8)	0.0979 (12)	0.0941 (12)	0.0176 (8)	0.0207 (8)	−0.0184 (9)
F3	0.0553 (9)	0.1373 (16)	0.1000 (13)	0.0088 (9)	0.0510 (10)	0.0268 (11)
O1	0.0360 (8)	0.0348 (8)	0.0701 (11)	−0.0005 (6)	0.0236 (8)	0.0001 (7)
O2	0.0343 (8)	0.0324 (8)	0.0613 (9)	0.0017 (6)	0.0186 (7)	0.0006 (7)
O3	0.0479 (10)	0.0503 (11)	0.0954 (14)	0.0101 (8)	−0.0043 (10)	0.0101 (10)
O4	0.0299 (7)	0.0383 (8)	0.0498 (8)	−0.0009 (6)	0.0162 (7)	0.0008 (6)
O5	0.0585 (10)	0.0714 (11)	0.0481 (9)	−0.0273 (8)	0.0332 (8)	−0.0229 (8)
O6	0.0413 (8)	0.0343 (7)	0.0486 (8)	−0.0027 (6)	0.0251 (7)	−0.0046 (6)
N1	0.0414 (10)	0.0327 (9)	0.0412 (10)	−0.0059 (7)	0.0227 (8)	−0.0031 (7)
N2	0.0370 (9)	0.0333 (9)	0.0484 (11)	0.0003 (7)	0.0188 (9)	0.0021 (8)
C1	0.0335 (11)	0.0333 (11)	0.0376 (11)	0.0014 (9)	0.0136 (9)	−0.0038 (9)
C2	0.0320 (10)	0.0342 (11)	0.0420 (12)	0.0002 (8)	0.0127 (9)	−0.0051 (9)
C3	0.0352 (12)	0.0414 (12)	0.0525 (14)	−0.0022 (9)	0.0133 (10)	0.0013 (10)
C4	0.0333 (12)	0.0398 (12)	0.0641 (16)	0.0040 (10)	0.0040 (11)	−0.0044 (11)
C5	0.0293 (11)	0.0587 (15)	0.0595 (15)	0.0078 (10)	0.0107 (11)	−0.0145 (12)
C6	0.0395 (13)	0.0749 (17)	0.0589 (15)	−0.0032 (12)	0.0272 (12)	−0.0021 (13)
C7	0.0368 (12)	0.0494 (13)	0.0493 (13)	0.0042 (10)	0.0171 (11)	0.0046 (10)
C8	0.075 (2)	0.0420 (15)	0.117 (3)	0.0082 (14)	0.0108 (19)	0.0039 (16)
C9	0.0517 (13)	0.0425 (12)	0.0446 (13)	−0.0117 (10)	0.0214 (12)	−0.0031 (10)
C10	0.0800 (19)	0.0488 (14)	0.0462 (14)	−0.0213 (13)	0.0281 (14)	−0.0070 (11)
C11	0.107 (2)	0.0386 (13)	0.0527 (15)	−0.0197 (13)	0.0503 (17)	−0.0102 (11)
C12	0.0781 (17)	0.0293 (11)	0.0666 (16)	−0.0064 (11)	0.0533 (15)	−0.0033 (11)
C13	0.0503 (13)	0.0271 (10)	0.0519 (13)	−0.0052 (9)	0.0329 (11)	−0.0020 (9)
C14	0.0447 (12)	0.0260 (10)	0.0625 (15)	0.0011 (9)	0.0336 (12)	0.0022 (9)
C15	0.0467 (14)	0.0363 (12)	0.0914 (19)	0.0067 (10)	0.0402 (15)	0.0103 (12)
C16	0.0457 (15)	0.0502 (15)	0.115 (3)	0.0113 (12)	0.0409 (17)	0.0192 (16)
C17	0.0377 (13)	0.0542 (15)	0.0792 (19)	0.0011 (11)	0.0080 (13)	0.0183 (14)
C18	0.0464 (14)	0.0460 (13)	0.0542 (15)	−0.0014 (11)	0.0142 (12)	0.0066 (11)
C19	0.078 (2)	0.0483 (15)	0.124 (3)	0.0137 (14)	0.080 (2)	0.0077 (16)
C20	0.098 (2)	0.0439 (14)	0.097 (2)	0.0037 (14)	0.078 (2)	−0.0019 (14)
F4	0.0363 (7)	0.1105 (12)	0.0457 (8)	−0.0016 (7)	0.0190 (6)	−0.0098 (8)
F5	0.0470 (9)	0.190 (2)	0.1187 (15)	0.0118 (11)	0.0517 (10)	−0.0143 (14)
F6	0.0335 (8)	0.1689 (19)	0.0909 (12)	0.0075 (9)	0.0129 (8)	0.0109 (12)
O7	0.0456 (9)	0.1017 (14)	0.0431 (10)	0.0044 (9)	0.0229 (8)	0.0051 (9)
O8	0.0359 (8)	0.0670 (10)	0.0460 (9)	0.0094 (7)	0.0193 (7)	−0.0012 (8)
O9	0.0612 (13)	0.260 (4)	0.0701 (14)	−0.0121 (17)	0.0444 (12)	−0.0422 (18)
C21	0.0371 (12)	0.0403 (12)	0.0441 (13)	−0.0032 (9)	0.0201 (10)	−0.0048 (9)
C22	0.0341 (11)	0.0355 (11)	0.0445 (12)	−0.0003 (8)	0.0185 (10)	0.0018 (9)
C23	0.0311 (11)	0.0537 (13)	0.0484 (13)	−0.0015 (9)	0.0182 (10)	−0.0026 (10)
C24	0.0470 (15)	0.089 (2)	0.0621 (16)	−0.0047 (13)	0.0322 (13)	−0.0105 (14)
C25	0.0389 (14)	0.091 (2)	0.081 (2)	0.0059 (13)	0.0361 (14)	−0.0008 (16)
C26	0.0289 (12)	0.0825 (18)	0.0704 (18)	0.0039 (12)	0.0144 (12)	0.0074 (14)
C27	0.0414 (13)	0.0652 (15)	0.0503 (14)	0.0001 (11)	0.0187 (11)	0.0034 (12)
C28	0.092 (3)	0.349 (8)	0.084 (3)	0.051 (4)	0.057 (2)	0.034 (4)

### Geometric parameters (Å, º)

**Table:**

Co1—O6	2.0931 (13)	C10—C11	1.354 (4)
Co1—O4	2.1016 (14)	C10—H10	0.9300
Co1—O2	2.1074 (14)	C11—C12	1.405 (4)
Co1—O5	2.1143 (14)	C11—H11	0.9300
Co1—N1	2.1290 (16)	C12—C13	1.411 (3)
Co1—N2	2.1652 (17)	C12—C20	1.427 (4)
F1—C3	1.353 (3)	C13—C14	1.434 (3)
F2—C5	1.348 (2)	C14—C15	1.406 (3)
F3—C6	1.358 (3)	C15—C16	1.395 (4)
O1—C1	1.256 (2)	C15—C19	1.447 (4)
O2—C1	1.250 (2)	C16—C17	1.361 (4)
O3—C4	1.370 (3)	C16—H16	0.9300
O3—C8	1.411 (3)	C17—C18	1.404 (3)
O4—H4A	0.8481	C17—H17	0.9300
O4—H4B	0.8494	C18—H18	0.9300
O5—H5A	0.8489	C19—C20	1.337 (4)
O5—H5B	0.8490	C19—H19	0.9300
O6—H6A	0.8476	C20—H20	0.9300
O6—H6B	0.8508	F4—C23	1.343 (2)
N1—C9	1.324 (3)	F5—C25	1.356 (3)
N1—C13	1.356 (3)	F6—C26	1.352 (3)
N2—C18	1.322 (3)	O7—C21	1.245 (2)
N2—C14	1.366 (3)	O8—C21	1.250 (2)
C1—C2	1.508 (3)	O9—C28	1.264 (4)
C2—C3	1.378 (3)	O9—C24	1.353 (3)
C2—C7	1.389 (3)	C21—C22	1.525 (3)
C3—C4	1.390 (3)	C22—C23	1.373 (3)
C4—C5	1.369 (3)	C22—C27	1.394 (3)
C5—C6	1.376 (3)	C23—C24	1.393 (3)
C6—C7	1.364 (3)	C24—C25	1.380 (4)
C7—H7	0.9300	C25—C26	1.373 (4)
C8—H8A	0.9600	C26—C27	1.369 (3)
C8—H8B	0.9600	C27—H27	0.9300
C8—H8C	0.9600	C28—H28A	0.9600
C9—C10	1.394 (3)	C28—H28B	0.9600
C9—H9	0.9300	C28—H28C	0.9600
			
O6—Co1—O4	176.42 (5)	C11—C10—C9	118.9 (2)
O6—Co1—O2	90.94 (5)	C11—C10—H10	120.5
O4—Co1—O2	88.54 (5)	C9—C10—H10	120.5
O6—Co1—O5	81.85 (6)	C10—C11—C12	120.3 (2)
O4—Co1—O5	94.63 (6)	C10—C11—H11	119.8
O2—Co1—O5	92.40 (6)	C12—C11—H11	119.8
O6—Co1—N1	88.89 (6)	C11—C12—C13	116.9 (2)
O4—Co1—N1	94.67 (6)	C11—C12—C20	124.5 (2)
O2—Co1—N1	92.83 (6)	C13—C12—C20	118.6 (2)
O5—Co1—N1	169.43 (6)	N1—C13—C12	122.4 (2)
O6—Co1—N2	98.62 (6)	N1—C13—C14	117.17 (17)
O4—Co1—N2	82.53 (6)	C12—C13—C14	120.5 (2)
O2—Co1—N2	166.20 (6)	N2—C14—C15	122.8 (2)
O5—Co1—N2	98.76 (7)	N2—C14—C13	117.61 (17)
N1—Co1—N2	77.52 (7)	C15—C14—C13	119.6 (2)
C1—O2—Co1	129.80 (13)	C16—C15—C14	117.1 (2)
C4—O3—C8	116.0 (2)	C16—C15—C19	124.8 (2)
Co1—O4—H4A	104.6	C14—C15—C19	118.1 (3)
Co1—O4—H4B	119.4	C17—C16—C15	120.2 (2)
H4A—O4—H4B	111.5	C17—C16—H16	119.9
Co1—O5—H5A	116.4	C15—C16—H16	119.9
Co1—O5—H5B	133.2	C16—C17—C18	119.2 (3)
H5A—O5—H5B	110.2	C16—C17—H17	120.4
Co1—O6—H6A	115.4	C18—C17—H17	120.4
Co1—O6—H6B	113.3	N2—C18—C17	122.7 (2)
H6A—O6—H6B	108.4	N2—C18—H18	118.7
C9—N1—C13	118.23 (18)	C17—C18—H18	118.7
C9—N1—Co1	127.37 (14)	C20—C19—C15	122.0 (2)
C13—N1—Co1	114.34 (13)	C20—C19—H19	119.0
C18—N2—C14	117.97 (18)	C15—C19—H19	119.0
C18—N2—Co1	128.57 (15)	C19—C20—C12	121.2 (2)
C14—N2—Co1	112.24 (13)	C19—C20—H20	119.4
O2—C1—O1	126.14 (18)	C12—C20—H20	119.4
O2—C1—C2	116.75 (17)	C28—O9—C24	126.8 (3)
O1—C1—C2	117.10 (17)	O7—C21—O8	125.34 (19)
C3—C2—C7	117.77 (19)	O7—C21—C22	115.47 (18)
C3—C2—C1	122.87 (18)	O8—C21—C22	119.18 (18)
C7—C2—C1	119.28 (18)	C23—C22—C27	117.68 (19)
F1—C3—C2	120.08 (18)	C23—C22—C21	124.19 (18)
F1—C3—C4	116.8 (2)	C27—C22—C21	118.12 (19)
C2—C3—C4	123.2 (2)	F4—C23—C22	121.42 (17)
C5—C4—O3	120.2 (2)	F4—C23—C24	115.07 (19)
C5—C4—C3	117.3 (2)	C22—C23—C24	123.5 (2)
O3—C4—C3	122.4 (2)	O9—C24—C25	125.8 (2)
F2—C5—C4	120.0 (2)	O9—C24—C23	117.4 (2)
F2—C5—C6	119.5 (2)	C25—C24—C23	116.8 (2)
C4—C5—C6	120.5 (2)	F5—C25—C26	118.2 (2)
F3—C6—C7	119.7 (2)	F5—C25—C24	120.8 (2)
F3—C6—C5	118.7 (2)	C26—C25—C24	121.0 (2)
C7—C6—C5	121.7 (2)	F6—C26—C27	120.4 (2)
C6—C7—C2	119.6 (2)	F6—C26—C25	118.5 (2)
C6—C7—H7	120.2	C27—C26—C25	121.1 (2)
C2—C7—H7	120.2	C26—C27—C22	120.0 (2)
O3—C8—H8A	109.5	C26—C27—H27	120.0
O3—C8—H8B	109.5	C22—C27—H27	120.0
H8A—C8—H8B	109.5	O9—C28—H28A	109.5
O3—C8—H8C	109.5	O9—C28—H28B	109.5
H8A—C8—H8C	109.5	H28A—C28—H28B	109.5
H8B—C8—H8C	109.5	O9—C28—H28C	109.5
N1—C9—C10	123.2 (2)	H28A—C28—H28C	109.5
N1—C9—H9	118.4	H28B—C28—H28C	109.5
C10—C9—H9	118.4		
			
O6—Co1—O2—C1	−162.24 (18)	C10—C11—C12—C20	178.5 (2)
O4—Co1—O2—C1	14.22 (18)	C9—N1—C13—C12	−2.8 (3)
O5—Co1—O2—C1	−80.36 (18)	Co1—N1—C13—C12	174.58 (15)
N1—Co1—O2—C1	108.82 (18)	C9—N1—C13—C14	178.83 (17)
N2—Co1—O2—C1	63.7 (3)	Co1—N1—C13—C14	−3.8 (2)
O6—Co1—N1—C9	−76.76 (17)	C11—C12—C13—N1	2.5 (3)
O4—Co1—N1—C9	102.89 (17)	C20—C12—C13—N1	−176.21 (19)
O2—Co1—N1—C9	14.12 (17)	C11—C12—C13—C14	−179.17 (18)
O5—Co1—N1—C9	−105.5 (4)	C20—C12—C13—C14	2.1 (3)
N2—Co1—N1—C9	−175.84 (17)	C18—N2—C14—C15	−0.9 (3)
O6—Co1—N1—C13	106.12 (13)	Co1—N2—C14—C15	−169.32 (16)
O4—Co1—N1—C13	−74.23 (13)	C18—N2—C14—C13	179.10 (18)
O2—Co1—N1—C13	−163.00 (13)	Co1—N2—C14—C13	10.6 (2)
O5—Co1—N1—C13	77.4 (4)	N1—C13—C14—N2	−4.9 (3)
N2—Co1—N1—C13	7.04 (13)	C12—C13—C14—N2	176.77 (17)
O6—Co1—N2—C18	96.75 (18)	N1—C13—C14—C15	175.11 (18)
O4—Co1—N2—C18	−79.82 (18)	C12—C13—C14—C15	−3.3 (3)
O2—Co1—N2—C18	−129.9 (3)	N2—C14—C15—C16	1.9 (3)
O5—Co1—N2—C18	13.75 (19)	C13—C14—C15—C16	−178.1 (2)
N1—Co1—N2—C18	−176.32 (19)	N2—C14—C15—C19	−178.05 (19)
O6—Co1—N2—C14	−96.30 (13)	C13—C14—C15—C19	2.0 (3)
O4—Co1—N2—C14	87.13 (13)	C14—C15—C16—C17	−1.4 (3)
O2—Co1—N2—C14	37.0 (3)	C19—C15—C16—C17	178.5 (2)
O5—Co1—N2—C14	−179.31 (13)	C15—C16—C17—C18	0.0 (4)
N1—Co1—N2—C14	−9.37 (13)	C14—N2—C18—C17	−0.7 (3)
Co1—O2—C1—O1	−19.6 (3)	Co1—N2—C18—C17	165.64 (17)
Co1—O2—C1—C2	159.01 (13)	C16—C17—C18—N2	1.1 (4)
O2—C1—C2—C3	150.4 (2)	C16—C15—C19—C20	−179.5 (2)
O1—C1—C2—C3	−30.9 (3)	C14—C15—C19—C20	0.5 (4)
O2—C1—C2—C7	−32.7 (3)	C15—C19—C20—C12	−1.7 (4)
O1—C1—C2—C7	146.1 (2)	C11—C12—C20—C19	−178.3 (2)
C7—C2—C3—F1	176.35 (19)	C13—C12—C20—C19	0.4 (3)
C1—C2—C3—F1	−6.7 (3)	O7—C21—C22—C23	−162.0 (2)
C7—C2—C3—C4	−2.2 (3)	O8—C21—C22—C23	18.6 (3)
C1—C2—C3—C4	174.8 (2)	O7—C21—C22—C27	16.9 (3)
C8—O3—C4—C5	−115.6 (3)	O8—C21—C22—C27	−162.5 (2)
C8—O3—C4—C3	68.5 (3)	C27—C22—C23—F4	−177.8 (2)
F1—C3—C4—C5	−177.9 (2)	C21—C22—C23—F4	1.2 (3)
C2—C3—C4—C5	0.7 (3)	C27—C22—C23—C24	0.3 (3)
F1—C3—C4—O3	−1.8 (3)	C21—C22—C23—C24	179.2 (2)
C2—C3—C4—O3	176.7 (2)	C28—O9—C24—C25	24.9 (6)
O3—C4—C5—F2	4.9 (3)	C28—O9—C24—C23	−157.9 (4)
C3—C4—C5—F2	−179.0 (2)	F4—C23—C24—O9	0.6 (4)
O3—C4—C5—C6	−175.1 (2)	C22—C23—C24—O9	−177.6 (3)
C3—C4—C5—C6	1.1 (4)	F4—C23—C24—C25	178.0 (2)
F2—C5—C6—F3	−0.5 (4)	C22—C23—C24—C25	−0.1 (4)
C4—C5—C6—F3	179.4 (2)	O9—C24—C25—F5	−2.3 (5)
F2—C5—C6—C7	178.8 (2)	C23—C24—C25—F5	−179.5 (3)
C4—C5—C6—C7	−1.3 (4)	O9—C24—C25—C26	177.7 (3)
F3—C6—C7—C2	179.0 (2)	C23—C24—C25—C26	0.6 (4)
C5—C6—C7—C2	−0.3 (4)	F5—C25—C26—F6	−0.3 (4)
C3—C2—C7—C6	1.9 (3)	C24—C25—C26—F6	179.6 (3)
C1—C2—C7—C6	−175.2 (2)	F5—C25—C26—C27	178.9 (3)
C13—N1—C9—C10	0.8 (3)	C24—C25—C26—C27	−1.2 (5)
Co1—N1—C9—C10	−176.24 (16)	F6—C26—C27—C22	−179.5 (2)
N1—C9—C10—C11	1.5 (3)	C25—C26—C27—C22	1.3 (4)
C9—C10—C11—C12	−1.7 (3)	C23—C22—C27—C26	−0.8 (3)
C10—C11—C12—C13	−0.2 (3)	C21—C22—C27—C26	−179.8 (2)

### Hydrogen-bond geometry (Å, º)

**Table:**

*D*—H···*A*	*D*—H	H···*A*	*D*···*A*	*D*—H···*A*
O4—H4*A*···O1	0.85	1.78	2.604 (2)	163
O4—H4*B*···O8^i^	0.85	1.89	2.729 (2)	169
O5—H5*A*···O8^ii^	0.85	1.94	2.791 (2)	176
O5—H5*B*···O6^iii^	0.85	2.15	2.976 (2)	165
O6—H6*A*···O1^iv^	0.85	1.90	2.738 (2)	167
O6—H6*B*···O7^ii^	0.85	1.77	2.623 (2)	176

Symmetry codes: (i) *x*−1, *y*, *z*; (ii) −*x*+1, *y*+1/2, −*z*+3/2; (iii) −*x*, *y*−1/2, −*z*+3/2; (iv) *x*, *y*+1, *z*.
