# Supplementary figures and images for: Crystal structure of tri­aqua­(1,10-phen­anthroline-κ2 N,N′)(2,4,5-tri­fluoro-3-meth­oxy­benzoato-κO 1)cobalt(II) 2,4,5-tri­fluoro-3-meth­oxy­benzoate
Source: Acta Crystallogr Sect E Struct Rep Online. 2014 Oct 11;70(Pt 11):m367–8. doi: 10.1107/S1600536814022077 (PMC4257257; doi:10.1107/S1600536814022077)

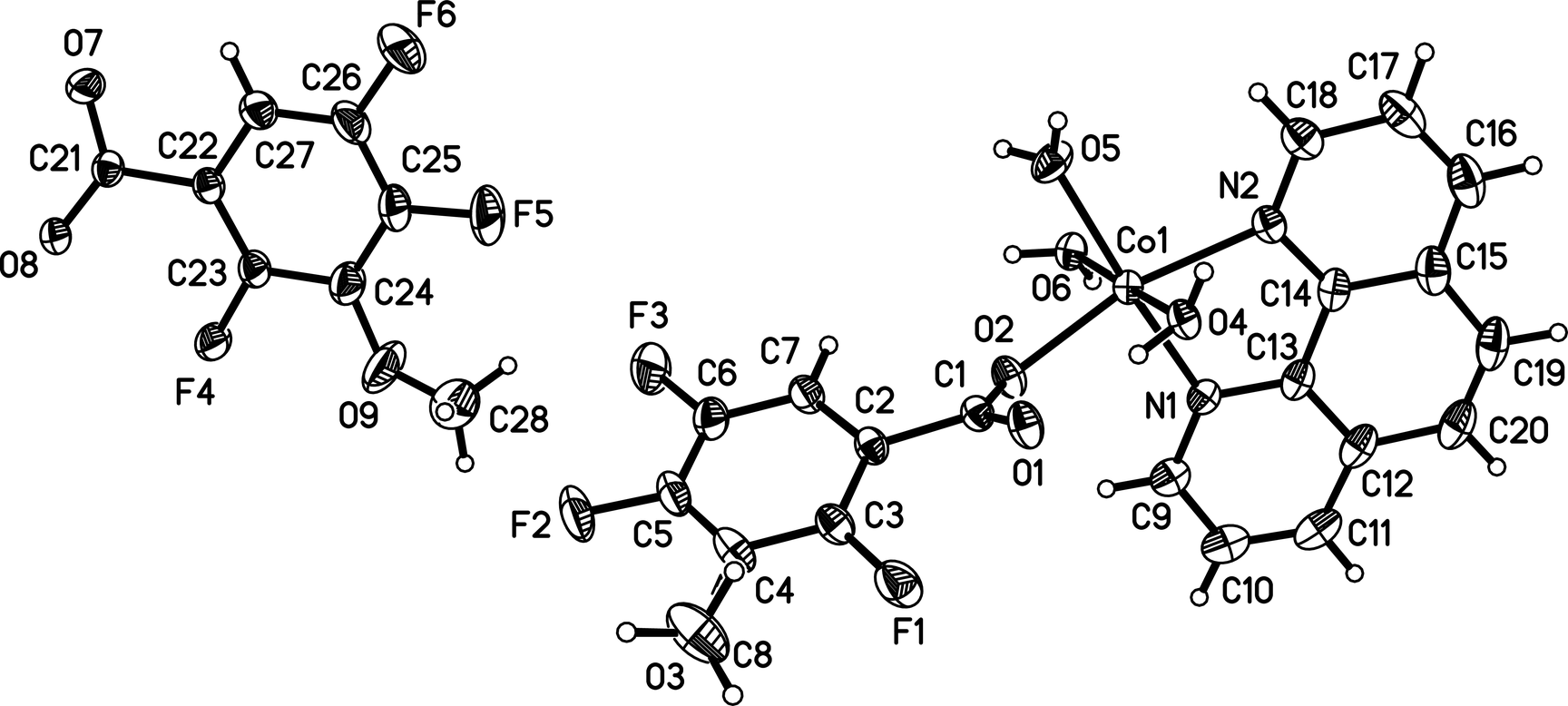

Supplement: Supplementary file 3 [file e-70-0m367-fig1.tif]

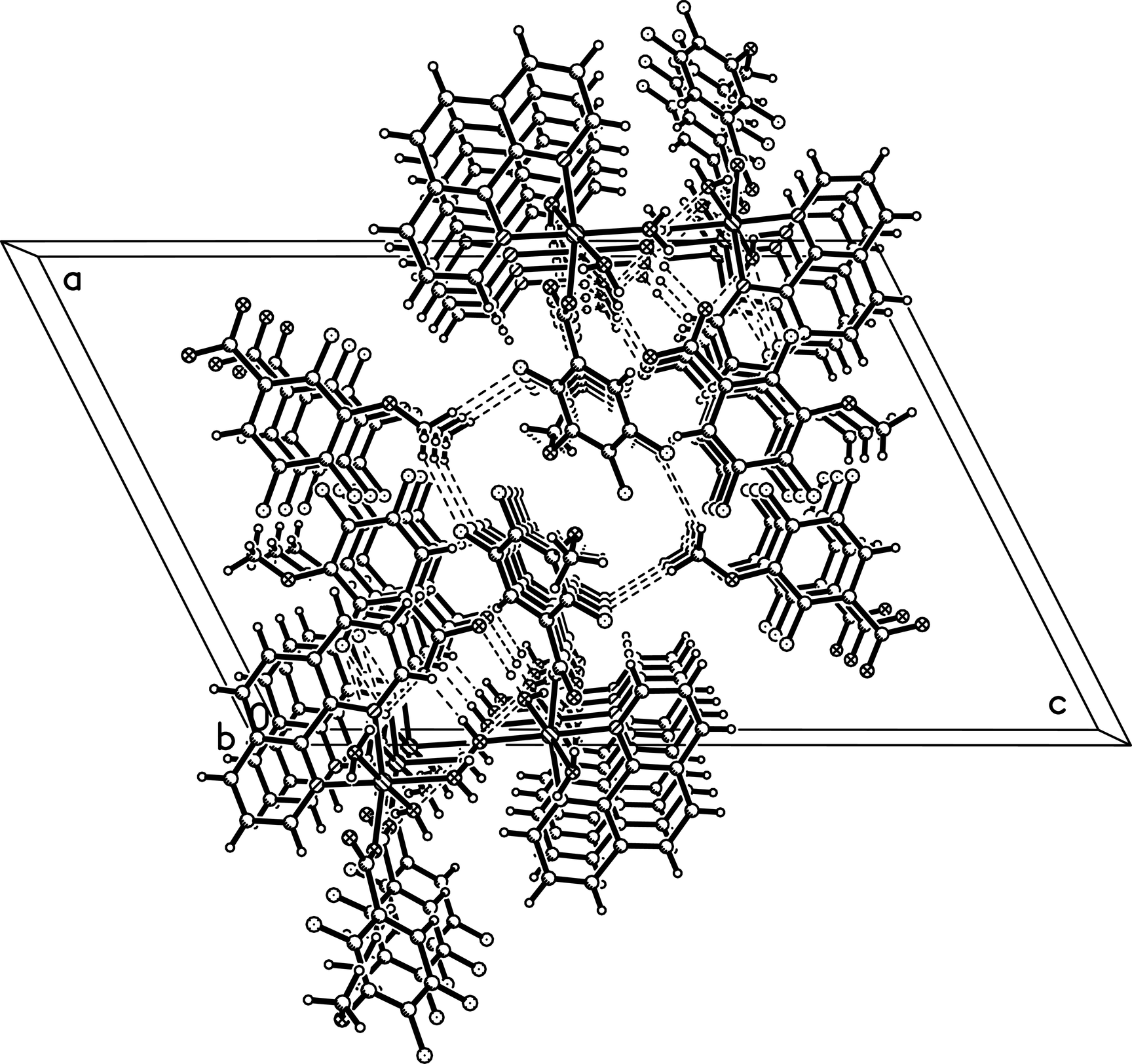

Supplement: Supplementary file 4 [file e-70-0m367-fig2.tif]
